# Application of Robotics in Orthodontics: A Systematic Review

**DOI:** 10.7759/cureus.58555

**Published:** 2024-04-18

**Authors:** Myriam Angélica De La Garza-Ramos, Héctor Hugo Ipiña-Lozano, Guillermo Cano-Verdugo, María Argelia Akemi Nakagoshi-Cepeda, Yinli Liu

**Affiliations:** 1 School of Dentistry, Universidad Autónoma de Nuevo León (UANL), Monterrey, MEX; 2 Department of Orthodontics, Academic Center for Dentistry (ACTA), Amsterdam, NLD

**Keywords:** systematic review, orthodontic wire bending, clinical application, orthodontics, robotics

## Abstract

Robotics has various applications in dentistry, particularly in orthodontics, although the potential use of these technologies is not yet clear. This review aims to summarize the application of robotics in orthodontics and clarify its function and scope in clinical practice. Original articles addressing the application of robotics in any area of orthodontic practice were included, and review articles were excluded. PubMed, Google Scholar, Scopus, and DOAJ were searched from June to August 2023. The risk of bias was established using the risk of bias in non-randomized studies (ROBINS) and certainty assessment tools following the grading of recommendations, assessment, development, and evaluation (GRADE) guidelines. A narrative synthesis of the data was generated and presented according to its application in surgical and non-surgical orthodontics. The search retrieved 2,106 articles, of which 16 articles were selected for final data synthesis of research conducted between 2011 and 2023 in Asia, Europe, and North America. The application of robotics in surgical orthodontics helps guide orthognathic surgeries by reducing the margin of error, but it does not replace the work of a clinician. In non-surgical orthodontics, robotics assists in performing customized bending of orthodontic wires and simulating orthodontic movements, but its application is expensive. The articles collected for this synthesis exhibited a low risk of bias and high certainty, and the results indicated that the advantages of the application of robotics in orthodontics outweigh the disadvantages. This project was self-financed, and a previous protocol was registered at the PROSPERO site (registration number: CRD42023463531).

## Introduction and background

The word “robot” was coined in 1920 by the Czech novelist Karel Capek [1], and its derived word “robotics” is considered to be an intelligent connection between perception and action. Robotics, comprising several scientific disciplines such as computer science and engineering, has significantly influenced various aspects of modern life, reflected in its contributions ranging from industrial manufacturing to medical care [2]. Robotics has played an active role in addressing the growing challenges presented by the medical sciences, specifically within the field of dentistry. Current literature has indicated that robots will be able to interact, explore, and work with humans, intervening in dental care and assistance [3].

In recent years, the robotics industry has shifted its vision toward autonomous robotic technology [4], facilitating minimally invasive techniques for certain operations [5]. In 2017, the completion of a dental treatment by a robot was reported [6-8], demonstrating the entrance of robots into various fields of dentistry [9]. Despite their relatively new application in orthodontics, robots have positioned themselves as a choice for performing routine activities that facilitate the work of an orthodontist [10].

No specific data are available regarding the number of orthodontists who employ robotics in their clinical practice; however, it is known that the global market for medical robots increased from $11.17 billion in 2022 to $13.25 billion in 2023. Currently, four types of robots are used in healthcare: surgical robots, exoskeletons, care robots, and hospital robots [11]. Considering the scarcity of current literature and the increasing use of robotics in oral healthcare, it is necessary to provide the scientific community with precise data regarding robotics in the orthodontics field. Thus, this systematic review was conducted to summarize the various applications of robotics while clarifying its role and scope in clinical practice.

## Review

Methodology

Statement Adherence

The Preferred Reporting Items for Systematic Reviews and Meta-Analyses (PRISMA) recommendations [12] were followed in this review.

Focused Question

What is the application of robotics in orthodontics?

Eligibility Criteria, Data Items, and Information Sources

From June to August 2023, an exhaustive search for research papers on the focused question was performed in PubMed, Google Scholar, Scopus, and Directory of Open Access Journals (DOAJ), omitting searches for gray literature. No age or language restrictions were established for the publications. The eligibility criteria and data items were chosen using the modification of the patient, intervention, comparison, outcome (PICO) framework [13]. Detailed information is presented in Table 1. Table 2 presents a comprehensive overview of the search strategy organized by data source.

**Table 1:** Inclusion and exclusion criteria and data items.

PICO element	Inclusion criteria	Exclusion criteria	Data items
P (Patient or population)	Any duty linked to the practice of orthodontics	Duties that do not represent a direct connection with orthodontics	Refers to the specialty of dentistry on which the study is focused
I (Intervention)	Application of machines for practical purposes in orthodontics	Manuscripts using artificial intelligence in orthodontics	Addresses the application of machines that assist orthodontic professional staff
O (Outcome)	Advantages and disadvantages of the application of robotics in orthodontics	Does not present the result of the practical contribution of robotics in orthodontics	The result of the application of technological devices that support the performance of orthodontic treatments
S (Study)	Only original articles	Review articles	Studies describing a real or practical application of robotics in orthodontics

**Table 2:** Search strategy.

Database	Search strategy	Articles retrieved
PubMed	(“robot”[All Fields] OR “robots”[All Fields] OR “robotically”[All Fields] OR “robotics”[MeSH Terms] OR “robotics”[All Fields] OR “robotic”[All Fields] OR “robotization”[All Fields] OR “robotized”[All Fields] OR “robots”[All Fields]) AND (“orthodontal”[All Fields] OR “orthodontic”[All Fields] OR “orthodontical”[All Fields] OR “orthodontically”[All Fields] OR “orthodontics”[MeSH Terms] OR “orthodontics”[All Fields])	99
Google Scholar	“robotic” AND “orthodontic” AND “dental”	1,930
Scopus	“robotic” AND “orthodontic”	69
DOAJ	“robotic” AND “orthodontic”	8

Data Extraction, Selection, and Collection Process

The data collected by the authors were organized into an Excel worksheet and categorized into the following columns: source with journal quartile ranking, authors, country, name or type of robots, application in orthodontics (surgical/non-surgical), objective, outcomes, conclusions, advantages, and disadvantages. The articles were selected by a methodical screening process, beginning with an initial assessment based on their titles, followed by a subsequent evaluation of the abstracts, and, ultimately, a thorough review of the full text. The searches were performed independently in PubMed and Scopus by the authors H.H.I.L. and in Google Scholar and DOAJ by G.C.V. Data collection was performed by the same author who searched the database and was verified by M.A.G.R. and M.A.A.N.C. for a consensus. In cases of disagreement, the support of Y.L. was requested.

Study Risk of Bias Assessment and Certainty Assessment

The risk of bias was established using the risk of bias in non-randomized studies (ROBINS) tool [14], with both individual and overall analyses. When data were lacking, the decision regarding whether to include an article was made by a group consensus. Regarding the certainty assessment, the grading of recommendations, assessment, development, and evaluation (GRADE) approach [15] was used to measure the quality of the evidence individually and collectively. Both processes were conducted by H.H.I.L. and Y.L.

Synthesis Methods

A narrative synthesis of the data was performed, and the data were categorized according to the use of robots in both surgical and non-surgical orthodontic treatments. Heterogeneity was evaluated according to the study design and the use of robotics in orthodontics.

Results

Study Selection

**Figure 1:**
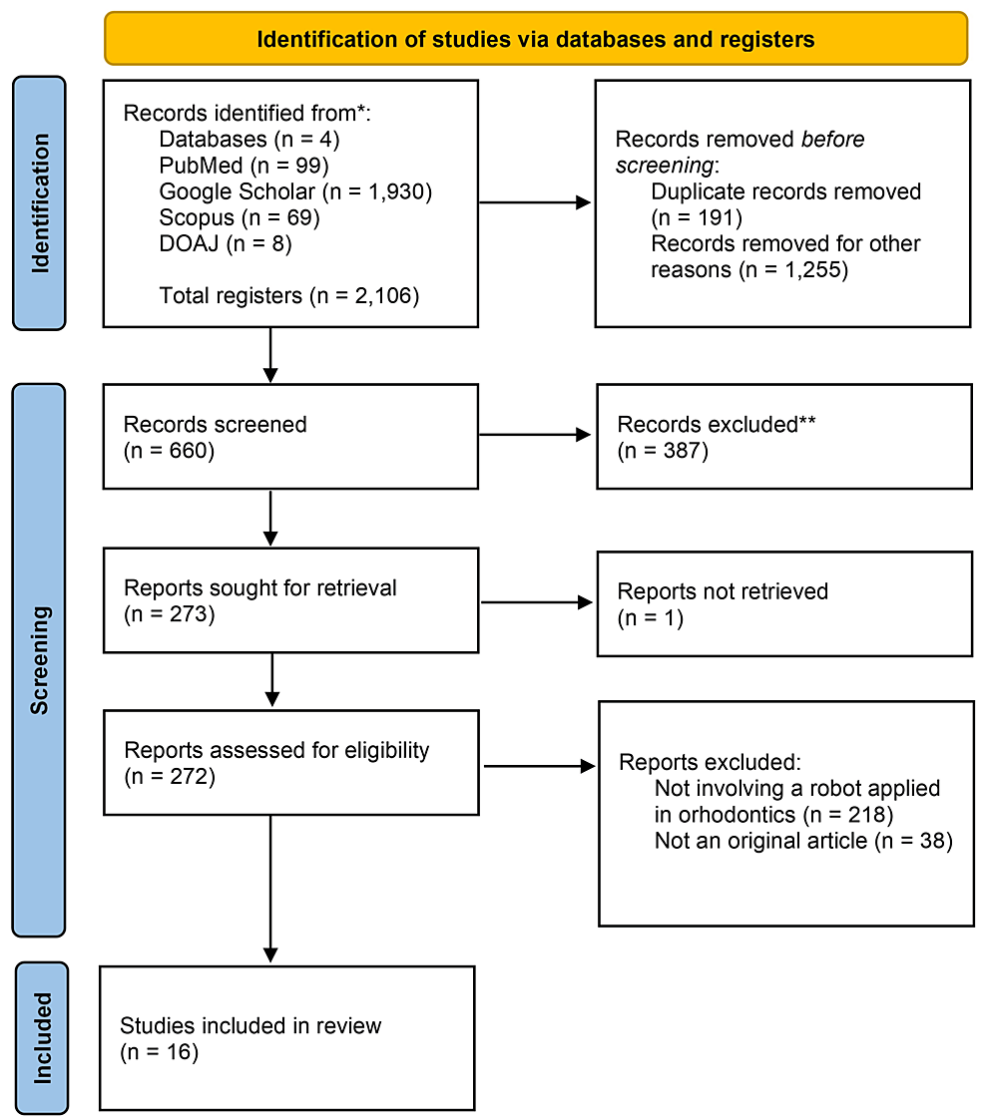
Study selection flowchart.

The literature search identified a total of 2,116 manuscripts, of which 191 were eliminated because they had been duplicated in the selected databases and 1,255 were eliminated after title analysis. Of the remaining 660 articles, 387 were eliminated after analyzing the abstract. In the end, 272 articles were selected for full-text analysis, and 16 articles were ultimately selected for final data synthesis corresponding to the main purpose of the study. Detailed information regarding the selection process is illustrated in Figure 1.

Study Characteristics and Results of Syntheses

The articles selected for synthesis comprised original research performed between 2011 and 2023. Although research oriented to this field is relatively new, the study of robotics has been observed to be gaining in popularity in Asia, Eastern Europe, and North America. Table 3 provides a summary of the obtained results.

**Table 3:** Main characteristics of the studies.

Source and journal quartile ranking	Author (s), date, country	Country	Name or type of robot	Application in orthodontics (surgical/non-surgical)	Objective	Outcomes	Conclusions	Advantages	Disadvantages
Google Scholar Q1	Jiang et al., 2017 [10]	China	Prototype of orthodontic archwire-bending robot	Not surgical	Calculate the elastic recovery of rectangular orthodontic archwires using a robot	The wire bending error decreased from 11.35% to 6.3% using the robot	The robot-operated system greatly improved the bending accuracy of the orthodontic archwire	Offers the possibility to standardize orthodontic wire-bending processes	Robot use could make manual labor obsolete
PubMed Q2	Ma et al., 2019 [16]	Japan	Oral and maxillofacial; name not specified	Surgical	Simulate oral and maxillofacial surgery in different jaw positions using a robot	The system has high precision in drilling bone in different mandibular positions	The system can successfully guide the completion of a maxillofacial operation	The robot used can reduce iatrogenic process-related orthodontic procedures	Disadvantages attributed to the robot attachments may induce poor z-axis detection
PubMed Q2	Wu et al., 2020 [17]	China	Robot-assisted orthognathic surgery; name not specified	Surgical	Compare errors in the accuracy and feasibility of robotically assisted orthognathic surgery and manual surgery	No significant differences were found in the errors of robotic and manual surgeries	The robot can operate orthognathic surgery as an adjunct to manual surgery	The robot is of great support in the precision of surgical cuts in operations	This robot is not a substitute for manual orthognathic surgery
PubMed Q1	Han et al., 2021 [18]	South Korea	Robot arm; name not specified	Surgical	Evaluate the feasibility and accuracy of a robotic arm combined with image-guided intraoperative navigation in orthognathic surgery	The robot may have great potential in precise repositioning of the jaws with minimal margins of error	The potential use of the robot in orthognathic surgery demonstrates high feasibility and precision	Supports real-time identification of anatomical structures, surgical planning, and verification of the surgical outcome	The system presents high cost, long preoperative time in software implementation structures, meticulous surgical planning, and fuzzy verification of the surgical outcome
PubMed Q2	Jiang et al., 2013 [19]	China	Multi-manipulator tooth-arrangement robot	Not surgical	Investigate robot control in orthodontic wire bending	The robot is capable of generating dental arches and bending orthodontic wires	The robot is capable of bending orthodontic wires	This system can be applied to different dental treatments and patient needs	Not specified
PubMed Q4	Gilbert et al., 2011 [20]	USA	LAMDA wire-bending robot	Not surgical	Compare bending of lingual orthodontic archwires by an office-operated robot to manual bending	Wire bending by the robotic system was positioned 2.1 points above manual bending	The robot proved to be successful in conducting lingual orthodontic treatments	The office-operated robot is simpler than those operated by technicians and reduces the possibility of errors	The robot requires a special computer program and only works on first-order arcs
PubMed Q1	Müller et al., 2016 [21]	Germany	CAD/CAM robot; name not specified	Not surgical	Determine the implementation accuracy of an office-operated robot in custom orthodontic wire bending	The accuracy of the robot was less than 0.5 degrees in rotation, inclination, and angulation movements, demonstrating (ddg1) better results in incisors and upper premolars	The robot can be successfully implemented in the clinic, ensuring high precision	The robot has high precision in translation and rotational motion	Robot accuracy may change depending on whether the bending wire is an upper or lower arch
PubMed Q2	Dotzer et al., 2023 [22]	Germany	KUKA KR 5-sixx R650 robot digital application	Not surgical	Compare the forces and movements of robot- and non-robot-controlled orthodontic dental archwires in an in vitro study	The arcs generated significantly greater forces and movements than non-robot-controlled arcs in intrusion, rotation, and angulation	The robot is effective in generating rotation and angulation movements but not in dental intrusion	The use of the robot makes it possible to predict preoperative results in a patient before treatment	Improper robot movements could lead to root resorption and periodontal overloading
Google Scholar Q1	Carossa et al., 2020 [23]	Italy	Bionic jaw motion robot	Not surgical	Use a robot to analyze jaw movements in an in vitro study	The system provides accurate registration and reproduction of the maxillomandibular relationship under static and dynamic conditions	This method could represent a valuable tool in orthodontics for clinical and research purposes	The system records and reproduces mandibular movements quickly and inexpensively compared to articulators	Not specified
DOAJ Q2	Sabbagh et al., 2024 [24]	Germany	HOSEA Robotic Device	Not surgical	Investigate the forces and moments during orthodontic en-bloc retraction using a robotic biomechanical simulation system	The system can foresee cases in which a periodontal problem could occur	The system is an option to prevent unwanted events in orthodontic treatment	The system offers advantages in cases with midline discrepancies	Torsional play between bracket and archwire is not controlled
DOAJ Q2	Hamrol et al., 2023 [25]	Poland	FANUC robot	Not surgical	Investigate the possibility of replacing manual belt grinding with robotic grinding	Robotic grinding showed better results compared to manual grinding	Robotic belt grinding is more efficient in quality and produces more consistent results than manual grinding	Robotic belt grinding presents high efficiency and precision	High economical expenditure
DOAJ Q2	Jin-Gang et al., 2018 [26]	China	Orthodontic archwire bending robot	Not surgical	Automatically acquire the dental arch curve and implement coordinated control of the dental arch generator	The dental arch generator can automatically generate a dental arch curve that fits a patient according to the patient’s jaw arch parameters	The curve formed by the outer surface of each tooth is smooth and clear, the interdental spacing is appropriate, and the position of each tooth is properly aligned	The robot is fast and effective	Not declared
Scopus Q1	McKay et al., 2023 [27]	USA	Aidin Robotics, Anyang, South Korea	Not surgical	Evaluate forces and moments generated during the extrusion of a maxillary central incisor with clear aligners with a robot	The robot demonstrated statistically significant differences when compared by materials and groups	The robot is a good measure to simulate future orthodontic results	Simulation provides higher dimensional accuracy and better-fitting aligners	Not declared
Scopus Q2	Engeler et al., 2020 [28]	Switzerland	Hexapod Paros; MiCos GmbH, Eschbach, Germany	Not surgical	Assess the torsional load transfer of various commercially available stainless steel wires used for fixed retainers	There is a range of 0.7%–3.7% in this torsional load transfer to the central incisor	This may explain the unexpected complications in multi-braid retainers	The robot can simulate the clinical situation before debonding	Not declared
Scopus Q1	Hu et al., 2022 [29]	China	Flexible six-dimensional force sensor	Not surgical	To develop a flexible six-dimensional force sensor for orthodontic treatments	The robot can be used in orthodontic treatment for precise correction with a full collection of orthodontic force	This unique flexible six-dimensional force sensor provides a new strategy for the design of multidimensional force sensors	This robot proposes novel materials and designs of micro/nanostructures based on piezoresistive	Not declared
Scopus Q1	Hsu et al., 2018 [30]	Taiwan	Water-powered soft actuator	Not surgical	Investigate the use of water-powered soft actuators for orthodontic application	This system is the first approach in designing a 3D scheme to predict the desired forces in orthodontics	This system is the first approach in designing a 3D scheme to predict the desired forces in orthodontics	When applied on teeth, osmotic actuators are expected to steadily overcome the rising resistance from surrounding issues	Force output and displacement rate may decrease over time

Application of Robotics in Surgical Orthodontic Treatment

Of 16 eligible articles, three focused on the use of robotics in surgical orthodontics, corresponding to studies performed in China, Korea, and Japan. Asia is the only continent on which this type of procedure is currently performed. The main purpose of all three manuscripts was to evaluate the use of robots in orthognathic surgery [16-18]. The primary benefits associated with the use of robots include their significant assistance to healthcare personnel in efficiently guiding jaw surgeries [17], reduction of iatrogenesis [16], facilitation of simple localization of anatomical structures, and increase in success rates within orthodontic treatment planning [18]. Additionally, robots allow the prediction of surgeries by simulating results, helping clinicians decide between the choices of orthodontic treatments with and without surgery [16].

Some disadvantages stated in these articles were the high costs, the long preoperative time spent using the software, and the lack of precision in detecting bone interferences. In all the reviewed cases, it was emphasized that robots do not replace clinicians in orthognathic surgery but rather serve as auxiliaries to the clinicians. None of these articles stated whether these robots should be used by the clinician or a technician specialized in the area [16-18].

Application of Robotics in Non-surgical Orthodontic Treatment

Of 16 eligible articles, 13 focused on the use of robotics in non-surgical orthodontics [10,19-30], corresponding to studies performed in Germany, Poland, Switzerland, Italy, China, Taiwan, and the United States. Among the articles selected for the synthesis, seven discussed the bending of orthodontic wires [10,19-21,25,26,28], while six addressed the use of robots in the simulation of dental and mandibular movements [22-24,27,29,30].

Regarding the literature on the use of robots in orthodontic wire bending, two studies proposed the use of a robot operated by the orthodontist in the office, without specifying whether prior training is required [21,22]. The remaining studies discussed devices operated by technical personnel. The robots are capable of bending traditional orthodontic and lingual orthodontic wires; however, it is noteworthy that some robots are only capable of bending archwires with a special shape such as rectangular and triangular, or archwires made of certain materials, such as nickel-titanium [10,19,19-22]. In contrast to the previous notion where disadvantages are stated regarding the employed robots, some studies exclusively declare advantages in their utilization [27,29].

In particular, a study by Engeler et al. [28], suggested wire bending in fixed retainers, a unique finding in this review. In this process, the authors highlighted the ability to predict torsional loads on certain teeth. All of the above cases presented the benefits of using this technology in the bending of orthodontic wires, which included favoring personalized treatments, reducing stress to the material by manual bending, and lessening working time. The stated disadvantages included the possible replacement of manual activities with robots, the need for a special computer program, the limitation to working on first-order arches [20], and changes to the accuracy of the robot depending on the area of focus [21].

Regarding the use of robots in the simulation of orthodontic treatments, robotics has been proposed as an alternative that can anticipate the final results of orthodontic treatments [22,27,29,30]. Nevertheless, Carossa et al. [23] reported that a jaw movement simulation robot can be used to analyze occlusal movements without requiring an articulator; in this case, the robot uses working models and replicates the patient’s jaw movements based on a previous scan. The disadvantages are that some robots can only be used for one type of material and their use is complex [10], they could cause root resorption and periodontal overload [22], and they could render manual work obsolete [10]. In contrast to the previous statement, in some studies, potential periodontal issues arising from orthodontic treatment were anticipated [24]. Other manuscripts included in the literature synthesis revealed the prediction of the forces required for torsional movements in orthodontics [25,26], and an article by McKay et al. [27] proposed a simulation for clear aligners. It is noteworthy that these last three studies report the presence of a special interface for the robot, a previously unreported detail.

Regarding the heterogeneity analysis, almost no heterogeneity was found concerning the study type, as only cross-sectional studies were identified focusing on the use of robots associated with orthodontic practice. The only reported difference could be attributed to the focus of robotics in treatments.

Risk of Bias and Certainty of Evidence

**Figure 2:**
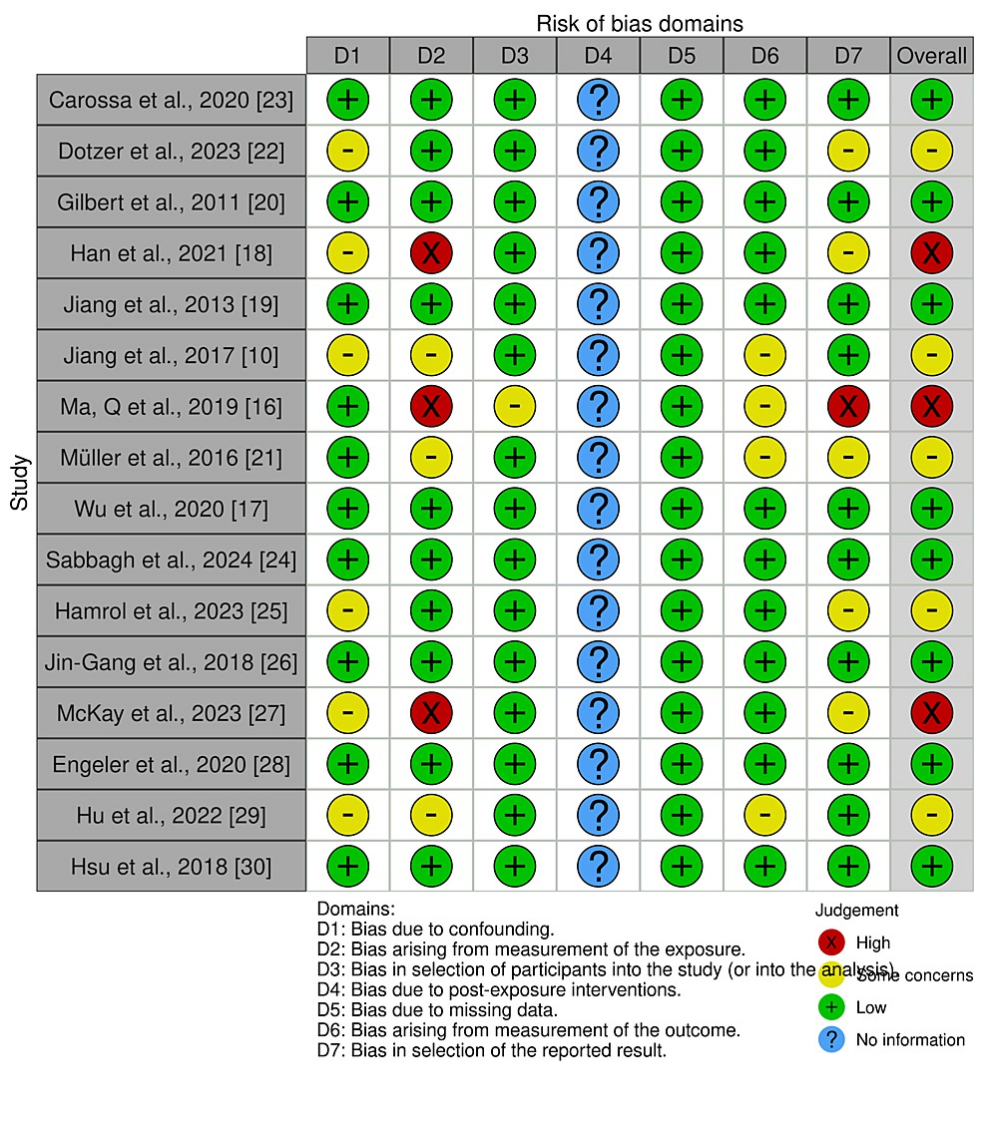
Results of risk of bias analysis per article.

**Figure 3:**
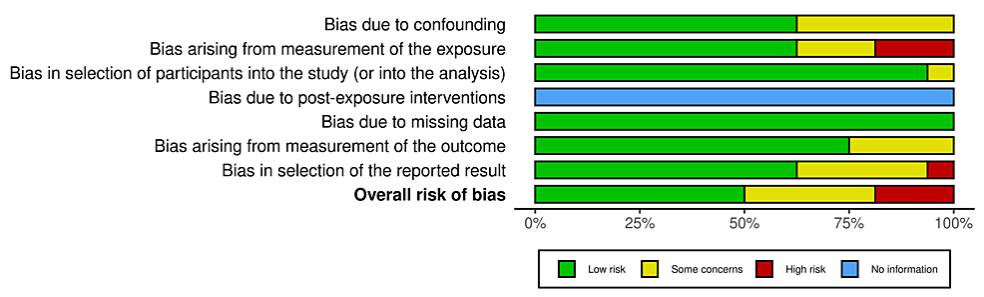
Results of the overall risk of bias analysis.

Analysis of the risk of bias produced the following results: 50% (8/16) of the studies reported low concerns about bias, 31.3% (5/16) reported some concerns, and 18.7% (3/17) reported high concerns. The detailed analysis by domain and manuscript is presented in Figure 2 and Figure 3. The overall analysis of the certainty of the evidence was high.

Discussion

The main purpose of this study is to describe the use of robotics in orthodontics. The results of the synthesis of the eligible articles indicate that robotics can be applied in orthodontics for both surgical and non-surgical purposes. The main advantages highlighted were a reduction in time and margin of error in the performance of the procedures while the disadvantages were high costs and requirements for specialized equipment and technical personnel to operate these devices.

This outcome is in agreement with that of Van Riet et al. [31], who highlighted that one of the fundamental interactions that robots have is with orthodontic wires. Adel et al. [32] also mentioned that the use of robots is considerably promising in the area of orthodontics. Supporting the benefits of robots in orthodontics, Liu et al. [33] stated that the use of robotics in dentistry helps to address the limitations and inadequacies of manual operations, resulting in finer and more precise movements that surpass what a human hand can achieve. Baxi et al. [34] made a similar observation, stating that some of the benefits of robots are reductions in treatment errors due to poor manipulation of appliances. In contrast to support for the use of robots in orthodontics, Li et al. [35] mentioned that limitations and challenges exist between research and the application of robots in clinical practice. Van Riet et al. [31] emphasized that a lack of scientific evidence exists regarding the benefits, outcomes, and cost-effectiveness of robots.

Continuing with the aforementioned, the data collected in this review demonstrate that dental clinical practice can experience significant benefits through the use of robots, owing to what is known as human-robot collaboration [36-39]. This collaboration spans from routine treatments to surgical procedures, thus expanding the spectrum of applications in the field of dentistry. Additionally, the use of artificial intelligence in combination with robots is highlighted, an aspect that has not been previously reported in this review [37]. According to recent research, this synergy between artificial intelligence and robotics significantly contributes to a comprehensive approach to medical treatment [38]. Specifically in the field of orthodontics, this combination can support both the clinician and the patient, thereby improving the quality of care and treatment outcomes [39].

The stated disadvantages are similar to those pointed out by Ahmad et al. [6], who argued that the limitations associated with robots are the difficult operating systems, high costs, complicated manipulation, and diffused sensory capabilities. These observations complemented those of Kumar et al. [40] and Jeelani et al. [41], both of whom highlighted the importance of evaluating the application of robotics in daily clinical practice, with consideration for how economical it can be.

The main strength of the present review is that it has encompassed all uses of robotics in orthodontics, highlighting advantages and disadvantages and positioning this study as a unique investigation of its kind. The main limitations of this study are that existing information was scarce and that the data provided by orthodontic associations were not considered. The results of this research can be used to support orthodontic practice in contexts in which treatment efficiency is required and the required infrastructure is available.

Regarding health policy, the results of this investigation can provide a solid basis for justifying the use of robots in surgical procedures. Future investigators interested in the subject can direct their research toward economic and feasibility analyses of the use of robotics in orthodontics and form multidisciplinary working teams, understanding that robotics stems from the engineering sciences.

## Conclusions

This systematic review explored the current use of robotics in the field of orthodontics, which represents a paradigm shift in the field, offering unprecedented precision and efficiency in various clinical procedures. The use of robotic technologies, such as computer-assisted diagnosis, automated treatment planning, and robotic-assisted surgeries, has demonstrated remarkable advancements in enhancing the overall quality of orthodontic care. These innovations not only streamline complex procedures but also contribute to improved treatment outcomes, reduced treatment durations, and enhanced patient comfort.

As the synergies between robotics and orthodontics continue to be explored and refined, it is evident that this convergence holds immense promise for revolutionizing traditional practices, ushering in a new era of personalized, technology-driven orthodontic interventions. The ongoing developments in this interdisciplinary domain underscore the transformative potential of robotics in reshaping the landscape of orthodontic treatment modalities.
